# Prevention of Esophageal Stricture After Whole Circumferential Endoscopic Resection: A Review for Endoscopists

**DOI:** 10.5152/tjg.2022.22298

**Published:** 2022-10-01

**Authors:** Jiale Zou, Ningli Chai, Enqiang Linghu, Zantao Wang, Longsong Li

**Affiliations:** 1 Medical School of The People’s Liberation Army (PLA), Beijing, China; 2 Department of Gastroenterology and Hepatology, The First Medical Centre, The People’s Liberation Army (PLA) General Hospital, Beijing, China

**Keywords:** Endoscopic resection, esophageal stricture, preventive measures

## Abstract

The incidence of esophageal stricture without stricture prophylaxis measures after whole circumferential endoscopic resection is almost 100%, which substantially decreases the patients’ quality of life and requires multiple sessions of endoscopic balloon dilation. To date, there are many reports concerning the prevention of esophageal stricture after whole circumferential endoscopic resection. Oral steroid may be effective for preventing esophageal stricture after whole circumferential endoscopic resection. However, exposure to a high dose of steroid raises concerns with regard to adverse events. Intralesional triamcinolone acetonide injection and preventive endoscopic balloon dilation did not appear to reduce the frequency of stricture formation after whole circumferential endoscopic resection. Esophageal stent appeared to be a possible prophylactic treatment, but adverse events should be of great concern. Polyglycolic acid sheets seemed promising, because they can not only act as protective barriers but can also be drug carriers to prevent esophageal stricture. Tissue engineering and regenerative medicine such as oral mucosal epithelial cell sheets cultured in vitro have been used in patients to prevent esophageal stricture, but it is technically and financially burdensome. Autologous tissue transplantation showed a promising preventive effect for esophageal stricture and it is relatively easy to carry out in clinical practice, and this technique needs further improvements to prevent esophageal stricture after whole circumferential endoscopic resection.

**Main Points**

- The incidence of esophageal stricture without stricture prophylaxis measures after whole circumferential endoscopic resection is almost 100%.
- Intralesional triamcinolone acetonide injection and preventive endoscopic balloon dilation did not appear to reduce the frequency of stricture formation after whole circumferential endoscopic resection.
- Autologous tissue transplantation showed a promising preventive effect for esophageal stricture and it is relatively easy to carry out in clinical practice.

## Introduction

**Figure 1.**
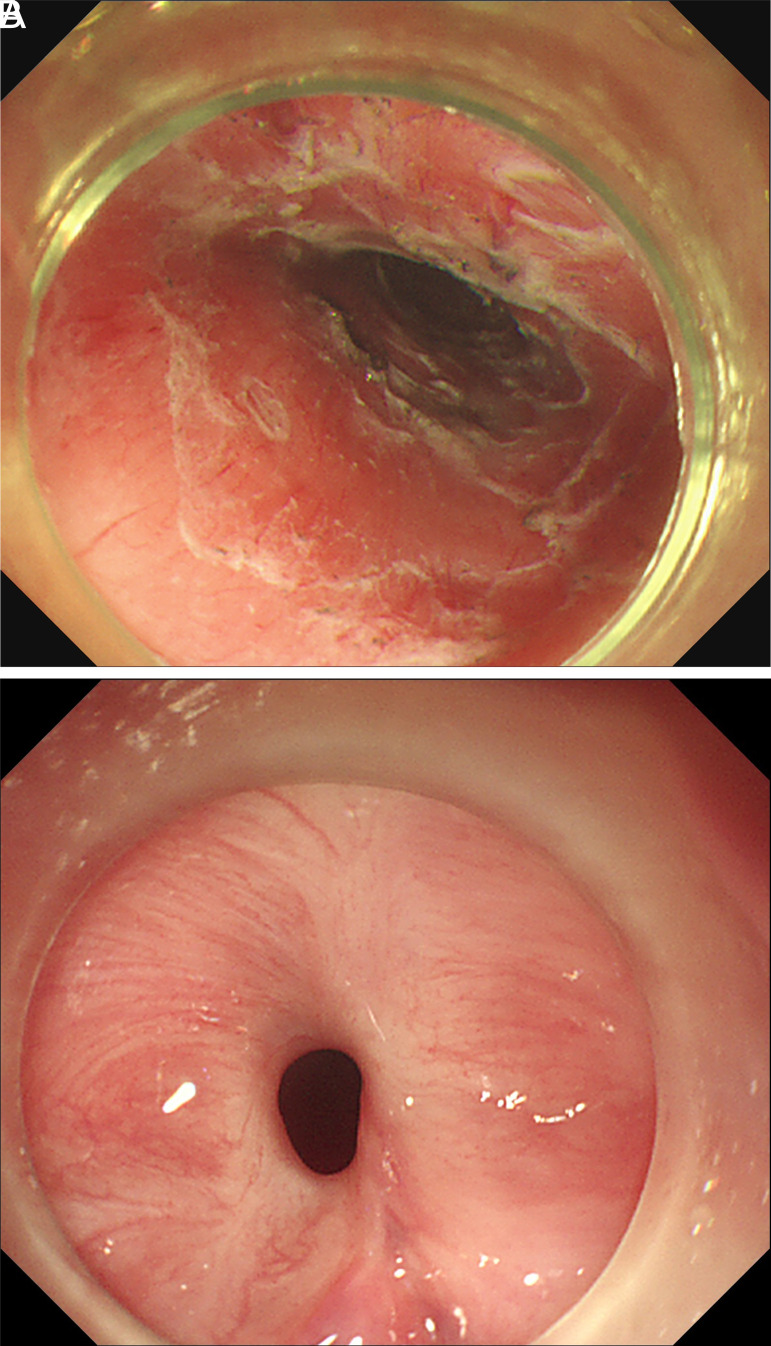
A case with extremely severe esophageal stricture after whole circumferential endoscopic submucosal dissection. (A) Endoscopic submucosal dissection resulted in a whole circumferential mucosal defect. (B) Three weeks after endoscopic submucosal dissection, the mucosal defect revealed a complete stricture that became pinhole-like.

Endoscopic resection (ER), including endoscopic mucosal resection (EMR) and endoscopic submucosal dissection (ESD), is widely accepted as a standard treatment for superficial esophageal neoplasms (SENs) because of its minimal invasiveness and good clinical outcomes.^1^ In particular, ESD allows the en bloc resection of lesions regardless of size, allowing accurate pathological evaluation and preventing local recurrence.^1,2^ Since the development of ESD, indications for ER have been gradually extended to large SENs. However, widespread mucosal dissection within the narrow esophageal lumen may result in severe stricture, which substantially decreases the patient’s quality of life and requires multiple sessions of endoscopic balloon dilation (EBD). For the whole circumferential ER, the postoperative stricture rate without stricture prophylaxis measures is almost 100%.^3-6^ In particular, whole circumferential ER is prone to refractory stricture despite the use of prophylactic measures.^7-9^ Although ESD is technically applicable for the en bloc resection of SENs involving the whole circumference, extremely severe stricture limits its widespread clinical application (Figure 1). To date, there are many reports concerning the prevention of stricture after ER for SENs. However, the included cases had different circular extents of resection, which prevented us from drawing conclusions on the efficacy and safety of prophylactic measures for preventing esophageal stricture after whole circumferential ER. Therefore, in this review, we aimed to focus on the current evidence on the prevention of postoperative stricture after whole circumferential ER.

### The Healing and Stricture Formation Process of Esophageal Mucosal Defects After Endoscopic Resection

For extensive esophageal mucosal defects after ER, stricture formation is a mucosal defect healing process. Most of our understanding of esophageal healing and stricture formation comes from animal models, which examine endoscopic findings and histological changes over time. These findings could be important for understanding the causes of esophageal stricture after extensive ER in the human esophagus and for devising a suitable treatment strategy.

In animal models of esophageal whole circumferential ER, esophageal mucosal defects were covered with white coats, and no obvious stricture was observed within 1 week after ER.^10^ Histologically, mucosal defects were extensively invaded by inflammatory cells.^11^ In the second week after ER, mucosal defects were covered with granulation tissue, and remarkable esophageal stricture was observed.^10,12,13^ Histologically, fibrous tissue hyperplasia accompanied by angiogenesis was observed in the submucosa.^10,11,13,14^ In addition, the regenerated squamous epithelium was observed at mucosal defect edges.^10,11,13-15^ It is worth noting that there were differences in epithelial regeneration between circumferential and non-circumferential esophageal mucosal defects. After circumferential resection, epithelial regeneration occurred only from the proximal and distal sides of defects, whereas after non-circumferential resection, epithelial regeneration occurred from the proximal side, distal side, and longitudinal residual normal mucosa.^16,17^ At 3-4 weeks after ER, whole circumferential mucosal defects revealed a complete stricture that became pinhole-like. Small esophageal mucosal defects heal and were covered with almost homogeneous squamous epithelium within approximately 1 month after ER.^11,15^ However, for large circumferential esophageal mucosal defects, histologic examination showed a lack of a continuous epithelial layer and a chronic, active inflammatory response even 8 weeks after ER.^18^ In addition, histological changes in the muscularis propria (MP) are also an important finding of the healing process after esophageal ER. Several studies have reported that the MP can undergo extensive destruction, and all layers of the MP were penetrated and replaced by fibrous tissue over time.^11,13,19^ However, Nonaka et al^10^ reported that there was no evidence of damage to the MP, but the thickness of the MP layer decreased with the loss of muscle fibers, and a layer of myofibroblasts appeared and increased in thickness over time.

The time course of the esophageal healing process after circumferential ER in animal models was mostly consistent with cases in the human esophagus, in which esophageal stricture developed at 2-3 weeks after whole circumferential ER.^4,6,11,14^ Mechanisms of esophageal stricture after extensive ER are complex. Extensive ER resulted in the loss of the barrier function of the epithelium, and the severe inflammation of mucosal defects occurred, which may be due to a combination of factors, including the passage of food and saliva, reflux of gastric acid and bile, microbes from the esophageal bacterial and fungal flora, and the side-effects of cauterization heat resulting from the use of high-frequency-wave electrosurgical instruments. Severe inflammation might gradually destroy the deeper layer of the esophagus and eventually lead to the fibrosis of the submucosa and atrophy of the MP, which could reduce the elasticity and compliance of tissue. In addition, considering the contractile ability of myofibroblasts, myofibroblasts may also play an active role in stricture formation.

### Steroid Administration

Steroids have been shown to attenuate the inflammatory process, reduce collagen synthesis and fibroblast proliferation, and promote fibroblast degeneration, thus inhibiting stricture formation.^20,21^ They have been routinely used to treat hypertrophic and keloid scars of the skin.^22^ To date, steroid administration is the most common treatment for the prevention of esophageal stricture after ER, and several meta-analyses have demonstrated that steroid administration effectively reduced the stricture rate and required EBD sessions.^23,24^ However, the optimal dose, duration, and administration form of steroids remain unclear (Table 1).

For oral steroid administration, Isomoto et al^25^ published a retrospective study including 7 patients who underwent whole circumferential ESD. Among these patients, 3 patients were treated with preemptive EBD, and the remaining 4 were administered oral prednisolone (PDN). Oral PDN was started at a dose of 30 mg daily on the third post-ESD day, gradually tapered (daily 30, 30, 25, 25, 20, 15, 10, 5 mg for 7 days each), and then discontinued 8 weeks later. Compared with the preemptive EBD group, the 4 patients in the oral PDN group required significantly fewer EBD sessions, of whom 2 patients did not experience esophageal stricture. Kataoka et al^26^ reported a short-period, low-dose protocol in which oral PDN was started with 30 mg daily on the second post-ESD day, continued with a gradual dose decrease (daily 30, 20, 10 mg for 7 days each), and then was discontinued 3 weeks later. One out of 3 patients treated with oral PDN after whole circumferential ESD developed esophageal stricture. Based on the current limited data, oral PDN may be effective for preventing esophageal stricture after whole circumferential ER, with stricture rates after oral PDN of 33%-50%.^25,26^ However, exposure to such a high dose of PDN raises concerns with regard to adverse events,^27^ including immunosuppression, optical damage, psychiatric disturbances, diabetes, peptic ulceration, and osteoporosis, especially for elderly patients with critical comorbidities and patients with uncontrolled diabetes or with previous infections.

Compared to oral steroid administration, intralesional steroid injection may have a lower risk of systemic adverse events owing to the small dose administered and short duration. To date, several studies have reported different intralesional steroid injection protocols for preventing esophageal stricture after ER. Kadota et al^4^ reported that intralesional triamcinolone acetonide (TA) injection was performed 3, 7, and 10 days after ER with 50 mg each time. Hashimoto et al^6^ reported that intralesional TA injection sessions were performed immediately after ESD and 14 days later, and the maximum dose of TA was 100 mg. The simplest protocol was reported by Takahashi et al,^5^ in which a single session of 40 mg TA was injected immediately after ESD, reducing the total dose of TA and eliminating the need for additional endoscopic interventions after ER. Based on the current data, intralesional steroid injection did not reduce the frequency of stricture formation after whole circumferential ER, and stricture rates after intralesional TA injection were as high as 80%-100%.^4-6,28^ An insufficient dose of TA and suboptimal interval of administration may be underlying reasons for the high stricture rate.

A more aggressive protocol of steroid administration for preventing esophageal stricture after ER involved oral PDN combined with intralesional TA injection. Kadota et al^4^ published a retrospective study including 25 patients who underwent whole circumferential ER. Among these patients, 5 patients had no prophylactic treatment, 6 patients underwent intralesional TA injection, and 14 patients underwent oral PDN combined with intralesional TA injection. The stricture rates were 100%, 100% and 71%, respectively. In regard to adverse events, 1 patient with oral PDN combined with intralesional TA injection was diagnosed with acute pneumonia 25 days after ESD. Iizuka et al^29^ also reported a combined steroid protocol for the prevention of esophageal stricture after whole circumferential ESD, in which the cumulative oral steroid dose exceeded 2000 mg combined with intralesional steroid injection, resulting in a stricture rate of 36.4% (4/11 patients), and adverse events such as candida esophagitis, arthritis, and steroid-related myopathy were observed.

In addition, some studies reported novel administration of steroids. Shibagaki et al^30^ applied the esophageal TA-filling method, in which the esophagus is filled with a saline solution with TA for a certain amount of time, allowing drug infiltration into the extensive resected surface. This study included 7 patients with whole circumferential ESD, and no patients required EBD during follow-up. Zhang et al^31^ reported oral administration of a mixture of hydrocortisone sodium succinate and aluminum phosphate for the prevention of esophageal stricture. This study included 6 patients with whole circumferential ESD and 3 patients with esophageal stricture during follow-up. These modified steroid methods showed a promising preventive effect for esophageal stricture, and multicenter studies with large sample sizes are needed to further evaluate these methods.

### Preventive Endoscopic Balloon Dilation

Traditional EBD has been used as a treatment for esophageal stricture after ER, whereas preventive EBD is performed before post-ER mucosal defects develop stricture (Table 2). Previous studies reported that preventive EBD was started 3 days to 1 week after ER and repeated once or twice a week for several weeks or until the complete healing of mucosal defects was observed.^3,25,32^ For patients with extensive mucosal defects involving over three-fourths of the lumen after ER, stricture rates after preventive EBD were 31.8%-68.8%.^3,32,33^ However, when analysis focused on whole circumferential ER, Yamaguchi et al^3^ reported that postoperative stricture occurred in all patients after receiving preventive EBD, and the mean number of EBD sessions was 32.7. In particular, 1 patient required as many as 48 sessions to relieve dysphagia, and the burden on the patient was great. Therefore, preventive EBD appeared to have no effect on the prevention of esophageal stricture after whole circumferential ER. Furthermore, the high incidence of esophageal perforation during EBD cannot be ignored and has been reported to occur in 0.4%-1.1% of EBD procedures for the treatment of esophageal stricture after ER.^34^

More recently, Li et al^35^ reported a novel self-help inflatable balloon that was passed through the patients’ nose into the esophagus to prevent esophageal stricture after whole circumferential ESD. This balloon could be easily operated by patients at home, starting on the fourth day after ESD and repeated 4-5 times every day until mucosal defects were almost healed. This study included 8 patients with whole circumferential mucosal defects, and only 1 patient experienced stricture after balloon removal. Currently, this was a single-center study, and adverse events such as sore throat and sore nose should be taken seriously.

### Esophageal Stents[Table t2-tjg-33-10-811]

The rationale of stenting to prevent esophageal stricture is to maintain esophageal patency through persistent radial force during the healing process, allowing the esophagus to remodel with an open lumen. Currently, there is no consensus regarding the type of stent, time of stent placement, and duration of stent placement in stricture prevention (Table 2).

To prevent esophageal stricture after whole circumferential ER, Ye et al^36^ placed a fully covered esophageal stent (FCES) immediately after ESD and removed it 3 months later. This prospective study included 23 patients, and 4 patients developed esophageal stricture (17.4%) during the follow-up period. Holt et al^37^ reported the application of an FCES 10 days after whole circumferential EMR for Barrett’s neoplasia, which was removed 8 weeks later, and 50% (6/12 patients) of patients ultimately developed esophageal stricture. Temporary FCES placement appeared to be a possible treatment for esophageal stricture after whole circumferential ER. However, the abovementioned studies involved a small sample size and had no control group; therefore, the results need to be confirmed by controlled studies with large samples. In addition, adverse events associated with metal stent placement, such as chest pain, stent migration, granulation tissue hyperplasia, and even life-threatening pseudoaneurysms,^16,37^ are of great concern.

Biodegradable stents are another potential option for the prevention of esophageal stricture. Currently, polydioxanone stents and poly-l-lactic acid stents are the two types of available biodegradable stents.^38-40^ Since biodegradable stents gradually degrade, there is no need to remove them. However, as the stent degrades, its efficacy in preventing esophageal stricture will gradually decrease. Saito et al^39^ reported that the early biodegradable stent migration rate was as high as 76.9% (10/13 patients). Yano et al^40^ reported that the long-term efficacy of biodegradable stents in the treatment of esophageal stricture after ESD was unsatisfactory, and none of the patients had improved dysphagia scores at 24 weeks after stent placement. For the prevention of esophageal stricture after whole circumferential ER, Pauli et al^41^ used a porcine model that demonstrated that biodegradable stent placement did not prevent esophageal stricture formation after whole circumferential mucosal resection. Therefore, the efficacy of biodegradable stents for the prevention of esophageal stricture after whole circumferential ER needs to be further verified.

### Polyglycolic Acid Sheets[Table t3-tjg-33-10-811]

Polyglycolic acid (PGA) sheets are a biodegradable suture material that has been demonstrated to prevent scar formation and contraction after glossectomy.^42^ Mechanisms underlying the prevention of esophageal stricture when using PGA sheets remain unknown. Polyglycolic acid sheets can act as barriers that protect mucosal defects from contact with exogenous materials, such as buccal secretions and food, thus inhibiting inflammation. In clinical practice, because PGA sheets easily fall off, fibrin glue is usually used to fix PGA sheets in post-ER mucosal defects (Table 3).

Because the efficacy of a single treatment with PGA sheets to prevent esophageal stricture after whole circumferential ER was limited, combination therapy involving PGA sheets to further improve outcomes was evaluated in some studies. Nagami et al^9^ evaluated the efficacy of intralesional TA injection and PGA sheets with fibrin glue for preventing esophageal stricture in patients after ESD, and stricture formation occurred in 4 of 6 (66.7%) patients with whole circumferential mucosal defects. Sakaguchi et al^43^ also assessed this combination therapy, in which postoperative stricture was successfully prevented in 1 out of 2 patients with whole circumferential mucosal defects. Chai et al^44^ conducted a randomized controlled study that demonstrated that PGA sheet-coated stent placement was more effective in preventing post-ESD esophageal stricture than stent placement alone. In this study, 14 patients with whole circumferential mucosal defects underwent PGA sheet-coated stent placement, and 6 patients (42.9%) experienced esophageal stricture. Li et al^45^ soaked PGA sheets in TA and then covered the stent with the PGA sheet, and stricture formation occurred in 3 of 6 (50%) patients with whole circumferential mucosal defects. Therefore, PGA sheets can not only act as protective barriers but can also be drug carriers to prevent esophageal stricture after ER. In addition, PGA sheets are highly advantageous in terms of safety because no adverse events have been reported to date. However, PGA sheets rapidly degrade, while the healing process of whole circumferential mucosal defects takes longer, and PGA sheets cannot act as protective barriers during the whole process of esophageal mucosal defect healing.

### Tissue Engineering and Regenerative Medicine

Tissue engineering and regenerative medicine have been used to prevent esophageal stricture after ER based on the concept that transplanted materials and tissue can repair and replace esophageal mucosal defects, thus ultimately reconstructing the structure and function of the esophagus.^46^ Cell-based therapy and extracellular matrix (ECM) scaffold-based therapy are the 2 main treatments for the prevention of esophageal stricture after ER. At present, most studies have been performed in animal models, and only a few have been used in a limited number of clinical facilities.

For cell-based therapy, autologous adipose tissue-derived stromal cells, autologous oral mucosal epithelial cells, and autologous epidermal cells have been used as alternative cell sources.^19,47-49^ The endoscopic injection of cell suspensions into the residual submucosa and the endoscopic transplantation of tissue-engineered autologous cell sheets are commonly used treatment strategies. Among these, autologous oral mucosal epithelial cell sheets have been used in patients to prevent esophageal stricture after ER. It usually takes approximately 2 weeks to culture cell sheets in vitro, and the diameter of the cell sheet is approximately 20 mm.^17,48,50,51^ The cell sheet retains cell membrane proteins and ECM, which can stably adhere to mucosal defects without the need for sutures or clips. Several clinical studies have indicated that the endoscopic transplantation of tissue-engineered autologous oral mucosal epithelial cell sheets can safely and effectively promote the re-epithelialization of the esophagus after extensive ER, decreasing both the risk and extent of stricture formation.^17,50,51^ However, based on the limited data, when analysis focused on whole circumferential mucosal defects, this technique did not reduce the frequency of stricture formation.^17,50,51^ It is worth noting that in current published clinical studies, for whole circumferential mucosal defects, the proportion of cell sheets covering mucosal defects was relatively low. If a large number of cell sheets can be cultured to increase the coverage of cell sheets of the whole circumferential mucosal defect, this might result in a lower frequency of stricture formation. Therefore, this technique needs to be further improved to prevent esophageal stricture after whole circumferential ER.

The ECM scaffold is made by a decellularization process that removes potentially immunogenic cellular material while retaining a 3-dimensional scaffold, which promotes tissue remodeling in the mucosal defect healing process.^18,52^ Currently, there are 3 main commercially available ECM products that have been used for the prevention of esophageal stricture after ER, namely, small intestine submucosa, acellular dermal matrix, and urinary bladder matrix. Unlike cell sheets, the placement of the ECM scaffold onto esophageal mucosal defects requires an esophageal stent or metal clips for fixation. Most studies were performed in animal models, and some of these studies have indicated that the endoscopic placement of an ECM scaffold prevented esophageal stricture after extensive ER.^18,52^ However, Schomisch et al^53^ reported that compared with stenting only, the addition of a commercially available ECM scaffold did not reduce esophageal stricture formation in swine models with 10-cm long whole circumferential mucosal defects after EMR. Hoppo et al^54^ applied an ECM scaffold created by decellularizing the porcine small intestinal submucosa onto mucosal defects after whole circumferential ER in 3 human patients. Among the 3 patients, 2 patients who had the complete coverage of the entire mucosal defect with ECM developed mild esophageal stricture, and another patient experienced tight stricture at the gastroesophageal junction where it had not been covered with ECM. Because ECM scaffolds are allogenic or xenogeneic and most studies have been performed in animal models; therefore, the safety and efficacy of ECM scaffolds in preventing esophageal stricture in humans remain unclear.

### Autologous Tissue Transplantation

Compared with tissue engineering approaches, the technique of autologous tissue transplantation is simpler because it does not require prior cell culture, cell sheet manipulation, or ECM generation. Considering the histological similarity and biological commonalities, appropriate transplanted tissue is applied with compression fixation on esophageal mucosal defects, absorbs fluid and nutritional components, and ultimately survives by vascularizing to esophageal mucosal defects. For the prevention of esophageal stricture, autologous tissue transplantation can not only protect mucosal defects but can also promote reepithelization (Table 4).

Hochberger et al^55^ reported a patient who underwent autologous gastric antral mucosa transplantation after whole circumferential ESD. The transplanted gastric mucosa grew well in the esophagus, and no evidence of stricture was observed. Liao et al^56^ reported autologous esophageal mucosal patch transplantation to mucosal defects using hemoclips and then fixed with an FCES in 9 patients who underwent whole circumferential ESD, and 8 of 9 patients experienced esophageal stricture. Zhang et al^57^ also applied this method, and 5 of 8 patients who underwent whole circumferential ESD experienced esophageal stricture. Recently, Liu et al^58^ used modified autologous esophageal mucosa transplantation, in which normal esophageal mucosal patches were sutured onto a PGA sheet with absorbable lines, and then the PGA sheet was sutured onto the FCES. With this modified method, no evidence of esophageal stricture was observed in a patient with a 10-cm long whole circumferential mucosal defect after ESD. In 2017, our team developed autologous skin-grafting surgery to prevent esophageal stricture after whole circumferential endoscopic submucosal tunnel dissection.^59,60^ The graft harvested from the outer thigh of the patient was sewn into “oversleeve-like” skin and then the skin graft was used to cover the outside of an FCES. Since the FCES itself also can prevent esophageal stricture, we conducted a case-matched controlled study^59^ that showed that autologous skin-grafting surgery was more effective in preventing esophageal stricture than FCES placement alone for whole circumferential mucosal defects.

The coverage rate and survival rate of transplanted tissue are the keys to its efficacy in preventing esophageal stricture. The selection of the donor site affects the amount of tissue harvested for transplantation. The esophageal lumen microenvironment, frequent esophageal peristalsis, and limited expansion force of the FCES had adverse impacts on the survival of transplanted tissue. Further improvements in autologous tissue transplantation are needed to prevent esophageal stricture.

## Conclusion

The healing pattern of whole circumferential esophageal mucosal defects is different from that of non-circumferential esophageal mucosal defects, which makes it difficult to prevent esophageal stricture after whole circumferential ER. Unlike other reviews that included cases with different circular extents of esophageal mucosal defects, our review focused on the current evidence on the prevention of postoperative stricture after whole circumferential ER. Currently, due to limited clinical data, no conclusive recommendation is clear for the prevention of esophageal stricture after whole circumferential ER. However, there have also been some developments for how endoscopists should manage patients after whole circumferential ER. First, endoscopists should fully inform patients of the extremely high risk of esophageal stricture after whole circumferential ER, preventive measures that can be taken, and the possible need for multiple dilations to treat stricture that occurs. Second, endoscopists should not directly copy the methods for preventing the post-ER stricture of non-circumferential esophageal mucosal defects to whole circumferential mucosal defects. For example, intralesional TA injection for non-circumferential esophageal mucosal defects showed favorable outcomes; however, it did not appear to reduce the frequency of stricture formation after whole circumferential ER. Third, combined treatment appeared to be more effective than a single treatment in the prevention of esophageal stricture after whole circumferential ER. For example, intralesional TA injection combined with PGA sheets or an FCES combined with PGA sheets could be used.

Tissue engineering and regenerative medicine for the prevention of esophageal stricture is promising, but it is technically and financially burdensome, and it needs to be further improved for the prevention of esophageal stricture after whole circumferential ER. Autologous tissue transplantation is a new technique for the prevention of esophageal stricture that is relatively easy to carry out in clinical practice. The selection of transplanted tissue and modification of the transplantation technique are needed to further develop this technique.

## Figures and Tables

**Table 1.** Studies on Steroid Administration for Prevention of Esophageal Stricture After Whole Circumferential Endoscopic Resection

Study	No. of Subjects	Protocol for Steroid Administration	Definition of Stricture	Stricture Rate	Adverse Events Related to Steroid
Kataoka M et al (2014)^26^	3	Oral PDN on the second day after ER; 30 mg, 20 mg, 10 mg for 7 days each	Dysphagia to solids or no passage of a 9.2 mm diameter endoscope	33.3% (1/3)	No
Isomoto H et al (2011)^25^	4	Oral PDN on the third day after ER; 30 mg, 30 mg, 25 mg, 25 mg, 20 mg, 15 mg, 10 mg, 5 mg for 7 days each	Not described	50% (2/4)	No
Kadota T et al (2016)^4^	6	Intralesional TA injection (50 mg) at 3, 7, and 10 days after ER	No passage of an endoscope (GIF Q260 or GIF 1T240)	100% (6/6)	No
Takahashi H et al (2015)^5^	5	Intralesional TA injection (40 mg) immediately after ER	Esophageal diameter <11 mm	100% (5/5)	No
Hashimoto S et al (2019)^6^	5	Intralesional TA injection (40-100 mg) immediately after ER and (16-50 mg) at 14 days after ER	Dysphagia to soft solids or no passage of an endoscope (GIF-Q240 or GIF-Q260J)	80% (4/5)	No
Funakawa K et al (2015)^28^	12	Intralesional TA injection immediately after ER, and at 1 week and 2 weeks after ER (the dose was not described)	Not described	83.3% (10/12)	No
Kadota T et al (2016)^4^	14	Intralesional TA injection + oral PDN; Intralesional TA injection (50 mg) at 3, 7, and 10 days after ER, oral PDN on the third day after ER (30 mg, 30 mg, 25 mg, 25 mg, 20 mg, 15 mg, 10 mg, 5 mg for 7 days each)	No passage of an endoscope (GIF Q260 or GIF 1T240)	71.4% (10/14)	1 case: acute pneumonia
Hanaoka N et al (2016)^7^	12	Intralesional TA injection + oral PDN; Intralesional TA injection (50-100 mg) immediately after ER, and oral PDN (5 mg/day) on the second day after ER and continued for 8 weeks	Dysphagia to some solids and no passage of a 9.2 mm diameter endoscope	91.7% (11/12)	No
Iizuka T et al (2018)^29^	11	Oral steroid a few days after ER; 30 mg, 30 mg, 25 mg, 25 mg, 20 mg, 15 mg, 10 mg, 5 mg for 7 days each; 6 of 11 cases also received intralesional TA injection (80-120 mg) after ER	No passage of an endoscope (GIF H260)	81.8% (9/11)	2 cases: pneumonia; 1 case: oral herpes infection
Iizuka T et al (2018)^29^	11	Oral steroid a few days after ER; 30 mg, 30 mg, 30 mg, 25 mg, 25 mg, 25 mg, 20 mg, 20 mg, 20 mg, 15 mg, 15 mg, 15 mg, 10 mg, 10 mg, 10 mg, 5 mg, 5 mg, 5 mg for 7 days each; 10 of 11 cases also received intralesional TA injection (80-120 mg) after ER	No passage of an endoscope (GIF H260)	36.4% (4/11)	7 cases: candida esophagitis; 1 case: arthritis; 2 cases: steroid-related myopathy
Kadota T et al (2020)^8^	26	Intralesional TA injection + oral PDN; Intralesional TA injection (50 mg or 100 mg) immediately after ESD, oral PDN on the third day after ESD (30 mg, 30 mg, 25 mg, 25 mg, 20 mg, 15 mg, 10 mg, 5 mg for 7 days each)	No passage of an endoscope (GIF Q260 or GIF 1T240)	61.5% (16/26)	No

PDN, prednisolone;

ER, endoscopic resection;

TA, triamcinolone acetonide.

**Table 2. t2-tjg-33-10-811:** Studies on EBD or Esophageal Stents for Prevention of Esophageal Stricture After Whole Circumferential Endoscopic Resection

Study	No. of Subjects	Interventions	Definition of Stricture	Stricture Rate
Yamaguchi N et al (2011)^3^	3	Preventive EBD was started on the third day post-ESD and continued twice weekly for 8 weeks	Not described	100% (3/3)
Li LS et al (2019)^35^	8	Self-help balloon was started on the fourth day post-ESD and repeated 4-5 times every day until mucosal defects were almost healed	No passage of an endoscope (GIF Q260)	12.5% (1/8)
Ye LP et al (2016)^36^	23	FCES placement immediately after ESD and removed it 3 months later	Not described	17.4% (4/23)
Holt BA et al (2016)^37^	12	FCES placement 10 days after EMR and removed it 8 weeks later	Dysphagia to some solids	50% (6/12)

EBD, endoscopic balloon dilation;

ESD, endoscopic submucosal dissection;

FCES, fully covered esophageal stent;

EMR, endoscopic mucosal resection.

**Table 3. t3-tjg-33-10-811:** Studies on PGA Combined Other Treatments for Prevention of Esophageal Stricture After Whole Circumferential Endoscopic Resection

Study	No. of Subjects	Interventions	Definition of Stricture	Stricture Rate
Nagami Y et al (2016)^9^	6	Intralesional TA injection (80 mg) + PGA sheets placement immediately after ESD	No passage of a 9.2 mm diameter endoscope	66.7% (4/6)
Chai NL et al (2018)^44^	14	PGA sheets + FCES placement immediately after ESD	Diameter of stricture section below 1 cm under endoscopy	42.9% (6/14)
Li LS et al (2019)^45^	6	TA-soaked PGA sheets + FCES placement immediately after ESD	Dysphagia to some solids and no passage of a 9.8 mm diameter endoscope	50% (3/6)
Sakaguchi Y et al (2016)^43^	2	Intralesional TA injection (40 mg) + PGA sheets placement immediately after ESD	No passage of 9-10 mm diameter endoscope	50% (1/2)

TA, triamcinolone acetonide;

PGA, polyglycolic acid sheets;

ESD, endoscopic submucosal dissection;

FCES, fully covered esophageal stent.

**Table 4.** Studies on Autologous Tissue Transplantation for Prevention of Esophageal Stricture After Whole Circumferential Endoscopic Resection

Study	No. of Subjects	Interventions	Definition of Stricture	Stricture Rate
Zou JL et al (2020)^59^	19	Autologous skin-grafting transplantation + FCES placement immediately after ESTD	No passage of a 9.8 mm diameter endoscope	36.8% (7/19)
Liao ZL et al (2018)^56^	9	Autologous esophageal mucosal patch transplantation + FCES placement immediately after ESD	Not described	88.9% (8/9)
Hochberger J et al (2014)^55^	1	Autologous gastric antral mucosa transplantation + uncovered metal stent placement immediately after ESD	Not described	0
Zhang Y, et al (2022)^57^	8	Autologous esophageal mucosal patch transplantation + FCES placement immediately after ESD	Not described	62.5% (5/8)
Liu Y et al (2020)^58^	1	Autologous esophageal mucosal patch transplantation + PGA sheet + FCES placement immediately after ESD	Not described	0

TA, triamcinolone acetonide;

PGA, polyglycolic acid sheets;

ESD, endoscopic submucosal dissection;

ESTD, endoscopic submucosal tunnel dissection;

FCES, fully covered esophageal stent.
